# Supplementary material for: The unspoken reality of gender bias in surgery: A qualitative systematic review
Source: PLoS One. 2021 Feb 2;16(2):e0246420. doi: 10.1371/journal.pone.0246420 (PMC7853521; doi:10.1371/journal.pone.0246420)
Supplement: S1 File — (PDF) [file pone.0246420.s002.pdf]

Supplementary File 1: Medline Search

1 exp Surgeons/
2 exp Specialties, Surgical/
3 (surger* or surgical or surgeon*).tw.
4 (neurosurgeon* or orthop?edic* or colorectal surgeon or general surgeon or gyn?ecologist or obstetrician or ophthalmologist or orthognathic surgeon or otolaryngologist or otorhinolaryngologist or plastic surgeon or thoracic surgeon or surgical oncologist or traumatologist or urologist).tw.
5 1 or 2 or 3 or 4
6 exp education, medical, graduate/
7 *education, medical/
8 (trainee* or training or resident* or residenc* or mentorship or postgraduate* or PGY*)
9 *Internship/ and Residency/
10 6 or 7 or 8 or 9
11 5 or 10
12 exp Sexism/
13 ((male or female or Sex or gender*) adj2 (bias* or discriminat* or inequality* or disparit* or prejudice* or attitude* or differen* or barrier* or sexis* or challenge* or issue* or problem* or barrier* or difficult* or hardship* or hard ship or concern*)).tw.
14 (sexis* or discriminat* or attrition or prejudice*).tw.
15 12 or 13 or 14
16 11 and 15
17 (((“semi-structured” or semistructured or unstructured or informal or “in-depth” or indepth or “face-to-face” or structured or guide) adj3 (interview* or discussion* or questionnaire*)) or (focus group* or qualitative or ethnograph* or fieldwork or “field work” or “key informant”)).tw. or interviews as topic/ or focus groups/ or narration/ or qualitative research/
18 16 and 17
19 limit 18 to english
