# Supplementary material for: The unspoken reality of gender bias in surgery: A qualitative systematic review
Source: PLoS One. 2021 Feb 2;16(2):e0246420. doi: 10.1371/journal.pone.0246420 (PMC7853521; doi:10.1371/journal.pone.0246420)
Supplement: S2 File — (PDF) [file pone.0246420.s003.pdf]

Supplementary File 2: CASP Qualitative Checklist

Paper	1. Was there a clear statement of the aims of the research?	2. Is a qualitative methodology appropriate?	3. Was the research design appropriate to address the aims of the research?	4. Was the recruitment strategy appropriate to the aims of the research?	5. Was the data collected in a way that addressed the research issue?	6. Has the relationship between researcher and participants been adequately considered?	7. Have ethical issues been taken into consideration?	8. Was the data analysis sufficiently rigorous?	9. Is there a clear statement of findings?	Overall assessment*
Hinze et al, 2004	Yes	Yes	Yes	Yes	Yes	No	Yes	Cannot tell	Yes	Minor concerns
Ozbilgin et al, 2011	Yes	Yes	Yes	Yes	Yes	Yes	Yes	Yes	Yes	No/very minor concerns
Brown et al, 2013	Yes	Yes	Yes	Yes	Yes	Yes	Yes	Yes	Yes	No/very minor concerns
Hill et al, 2015	Yes	Yes	Yes	Yes	Yes	Yes	Yes	Yes	Yes	No/very minor concerns
Rich et al, 2016	Yes	Yes	Yes	Yes	Yes	Yes	Yes	Yes	Yes	No/very minor concerns
Webster et al, 2016	Yes	Yes	Yes	Yes	Yes	Yes	Yes	Yes	Yes	No/very minor concerns
Dahlke et al, 2018	Yes	Yes	Yes	Yes	Yes	No	No	Yes	Yes	Moderate considerations

Myers et al, 2018	Yes	Yes	Yes	Yes	Yes	Yes	Yes	Yes	Yes	No/very minor concerns
Yi et al, 2018	Yes	Yes	Yes	Yes	Yes	Yes	Yes	Yes	Yes	No/very minor concerns
Barnes et al, 2019	Yes	Yes	Yes	Yes	Yes	Yes	Yes	Yes	Yes	No/very minor concerns
Bernardi et al, 2019	Yes	Yes	Yes	Yes	Yes	No	Yes	Yes	Yes	Minor concerns
Liang et al, 2019	Yes	Yes	Yes	Yes	Yes	Yes	Yes	Yes	Yes	No/very minor concerns
Lu et al, 2019	Yes	Yes	Yes	Yes	Yes	Yes	Yes	Yes	Yes	No/very minor concerns
Hutchison et al, 2020	Yes	Yes	Yes	Yes	Yes	Yes	Yes	Yes	Yes	No/very minor concerns

*The overall assessment was rated on a scale: ‘no/very minor concerns’, ‘minor concerns’, ‘moderate considerations’, and ‘serious concerns’
