# Supplementary material for: The unspoken reality of gender bias in surgery: A qualitative systematic review
Source: PLoS One. 2021 Feb 2;16(2):e0246420. doi: 10.1371/journal.pone.0246420 (PMC7853521; doi:10.1371/journal.pone.0246420)
Supplement: S3 File — (PDF) [file pone.0246420.s004.pdf]

Supplementary File 3: Standards for Reporting Quality Research (SRQR)

Paper	S1	S2	S3	S4	S5	S6	S7	S8	S9	S10	S11	S12	S13	S14	S15	S16	S17	S18	S19	S20	S21
Hinze et al, 2004		*	*	*			*	*	*	*	*	*	*	*		*	*	*	*		
Ozbilgin et al, 2011		*	*	*	*	*		*	*	*	*	*	*	*		*	*	*	*		*
Brown et al, 2013	*	*	*	*	*	*	*	*	*	*	*	*	*	*	*	*	*	*	*	*	
Hill et al, 2015		*	*	*	*	*	*	*	*	*	*	*	*	*	*	*	*	*	*	*	*
Rich et al, 2016	*	*	*	*			*	*	*	*	*	*	*	*	*	*	*	*	*	*	*
Webster et al, 2016	*	*	*	*	*	*	*		*	*	*	*	*	*	*	*	*	*	*		
Dahlke et al, 2018	*	*	*	*				*	*	*	*	*	*	*	*	*	*	*	*		
Myers et al, 2018	*	*	*	*	*	*	*	*	*	*	*	*	*	*	*	*	*	*	*	*	*
Yi et al, 2018	*	*	*	*	*	*	*	*	*	*	*	*	*	*	*	*	*	*	*	*	*
Barnes et al, 2019	*	*	*	*	*	*	*	*	*	*	*	*	*	*		*	*	*	*		*
Bernardi et al, 2019	*	*	*	*	*	*	*	*	*	*	*	*	*	*	*	*	*	*	*	*	*
Liang et al, 2019	*	*	*	*	*	*	*	*	*	*	*	*	*	*	*	*	*	*	*	*	*
Lu et al, 2019	*	*	*	*	*	*	*	*	*	*	*	*	*	*	*	*	*	*	*	*	*
Hutchison et al, 2020	*	*	*	*	*		*	*	*	*	*	*	*	*	*	*	*	*	*	*	*

*Represents standard was fulfilled

S1 Title
S2 Abstract
S3 Problem formulation
S4 Purpose or research question
S5 Qualitative approach and research paradigm
S6 Researcher characteristics, reflexivity
S7 Context
S8 Sampling strategy
S9 Ethical issues pertaining to human subjects
S10 Data collection methods
S11 Data collection instruments/ technologies
S12 Units of study
S13 Data processing
S14 Data analysis
S15 Techniques to enhance trustworthiness
S16 Synthesis and interpretation
S17 Links to empirical data
S18 Integration with prior work, implications, transferability and contribution(s)
S19 Limitations
S20 Conflicts of interest
S21 Funding
