# Supplementary material for: The unspoken reality of gender bias in surgery: A qualitative systematic review
Source: PLoS One. 2021 Feb 2;16(2):e0246420. doi: 10.1371/journal.pone.0246420 (PMC7853521; doi:10.1371/journal.pone.0246420)
Supplement: Attachment — Submitted filename: Response to reviewers.docx [file pone.0246420.s005.docx]

Dear Reviewers,

PLOS ONE

Subject: Submission of revised paper Ms. No. PONE-D-20-37354, titled “The unspoken reality of gender bias in surgery: A qualitative systematic review”.

We thank the reviewers for their comments and have amended the manuscript to address all their concerns accordingly. For ease of reference to the changes made, we have tabulated them below and highlighted them in our manuscript. We hope that the revised version will be suitable for publication. We look forward to hearing from you again.

| **Comment** | **Reviewer’s Comments** | **Response** | **Line Number** |
| --- | --- | --- | --- |
| **Reviewer #1** | | | |
| 1 | There are several large-size studies on gender bias (Ann Thorac Surg 2020; 109(1):14-17. n=663), sexual harassment (Ann Thorac Surg 2020; 109(4):1283-1288. n=790), and salary disparities (Ann Thorac Surg 2020; S0003-4975(20)31687-8. n=1069) in cardiothoracic surgery. Including these references would help to amplify your message. | Dear Reviewer, thank you for the comment. We have amended the manuscript to include the mentioned reference. | 59 |
| 2 | On the topic of motherhood and work-life balance, did the authors note the manuscript of gender differences in academic surgery and work-life balance of 127 faculty and 116 trainees (J Surg Res 2017; 218:99-107). Inclusion of this reference also would contribute significantly to the message of this manuscript. | Dear Reviewer, thank you for the comment. We have amended the manuscript to include the mentioned reference. | 59 |
|  | Line 74 (“gender disparities that cripple the progression of female surgeons.” Use of the word “cripple” is overly dramatic and the word renders a negative reaction. Making overstatements, such as this one, may turn away the audience and, hence, the message of this very important manuscript is lost. Recommend using “stunts” or “impedes” or a more neutral term that conveys the same thought. | Dear Reviewer, thank you for the comment. We have corrected the word choice to a more neutral one. | 75 |
| 3 | Line 224. “Camaraderie” is spelled incorrectly. | Dear Reviewer, thank you for the comment. We have corrected the spelling. | 225 |
| **Reviewer #2** | | | |
| **Major Issues** | | | |
| 1 | Confusion of sex and gender throughout the text  When you are talking about gender blindness he specifically state sex differences but I think your paper is about gender differences since it is societal roles and perceptions not biology that is determining the themes that you are addressing. Again, I think the language in this paper around sex and gender is mixed up- please consult Sager guidelines. | Dear Reviewer, thank you for the comment. We have clarified the respective terms and added in a clearer definition of gender which is the main scope of our paper into the discussion. | 273 - 274, 279, 303 – 306, 327 |
| 2 | Lack of clarity and whose voice is being represented when talking through the results  Be clear about whether you are stating a fact or the perceptions of the respondents in the qualitative studies. For example lines 179/180 Women were promoted at a slower pace or passed over in favour of male colleagues for referrals despite being equally qualified [25, 26, 33]. You have not made it clear whether this is the perception of individuals or based on data. I realize I could go look up each of these references and try to figure out what they say but I think you have the responsibility of being clear whether you are conveying perceptions from the qualitative literature which represent the thoughts and experiences of individuals or whether you are looking at quantitative data that reflects an analysis of what is happening. They may or may not be the same. Similarly it is often not clear whose voice you are representing from the qualitative literature. Example: others who had more familial duties were also perceived to be less committed to work, especially if they worked part time, and henceforth, regarded as less respectable… Lines 192/193… It is not clear whether it is qualitative analysis of the perceptions of supervising physicians and staff about the women surgeon,,, or it is the women surgeon’s perceptions of how they think the physicians and staff receive them… Again potentially related but potentially not and it is very important in qualitative research to be clear about whose views are being represented. I assume that you have included studies that have different perspectives and the perspective must be clear both in your chart of the included studies and when you are representing themes. Another example - female surgeons were also preferred and actively chosen by some patients for reasons including more delicate surgical work or being better equipped to understand and look after pregnant patients [32]…. It is not clear if you are analysing the perspectives of patients or how surgeons think patients have interacted with them. | Dear Reviewer, thank you for the comment. We have amended the results section to better reflect the statements of the respondents. | 173 - 174, 180, 185, 191 – 206, 212 – 213, 220 – 221, 239 – 240, 246 – 248, 268, 270 – 271, 278 – 279, 283 – 284, 297 |
| **Minor and editorial issues** | | | |
| 1 | Please do not start sentences with numbers unless you spell them out. | Dear Reviewer, thank you for the comment. We have amended the abstract accordingly. | 39 |
| 2 | The methods are sparsely described in the abstract into should include key issues like how many people screened/extracted data and how synthesis was completed. | Dear Reviewer, thank you for the comment. We have added more details into the methods section of the abstract. | 34 - 36 |
| 3 | In your introduction you state that gender discrimination has thwarted interest from women candidates and that is why women are underrepresented - what about bias in selection of candidates you know if the success rates are similar in terms of admission. | Dear Reviewer, thank you for the comment. We have added the relevant portions in the introduction. | 63 |
| 4 | The paper talks about 2 genders men and women but ignores gender diverse populations and how they have been treated in surgery. It may be this is due to a lack of literature or a lack of intention of addressing this issue- please clarify. | Dear Reviewer, thank you for the comment. We have added this into the limitations. | 461 - 463 |
| 5 | Start your discussion with an overall summary of the key contribution of the work in terms of findings not a statement of claim … this is the first qualitative systematic review …. | Dear Reviewer, thank you for the comment. We have amended the discussion to reflect this change. | 300 - 302 |
| 6 | It is certainly true that there is an underrepresentation of women but there is as you point out considerable efforts to change this and I think it would be good not only to say that underrepresentation exists but to have some data in your paper about how quickly this is changing. In other words are we on track for even representation in their near future or is the rate of change so slow that this underrepresentation is still not resolving. | Dear Reviewer, thank you for the comment. We have amended the discussion to reflect this change. | 379 - 383 |
